# Co-targeting translation and proteasome rapidly kills colon cancer cells with mutant *RAS/RAF* via ER stress

**DOI:** 10.18632/oncotarget.14063

**Published:** 2016-12-21

**Authors:** Xiangyun Li, Mei Li, Hang Ruan, Wei Qiu, Xiang Xu, Lin Zhang, Jian Yu

**Affiliations:** ^1^ First department, State Key Laboratory of Trauma, Burn and Combined Injury, Research Institute of Surgery and Daping Hospital, Third Military Medical University, Daping, Yu Zhong District, Chongqing 400042, P.R. China; ^2^ Department of Animal Genetics, Breeding and Reproduction, Nanjing Agricultural University, Weigang, Nanjing 210095, P.R. China; ^3^ Department of Pathology, University of Pittsburgh Cancer Institute, Pittsburgh, PA 15213, USA; ^4^ Department of Pharmacology and Chemical Biology, University of Pittsburgh School of Medicine, University of Pittsburgh Cancer Institute, Pittsburgh, PA 15213, USA

**Keywords:** translation, ER stress, CHOP, DR5, Bax

## Abstract

Colorectal cancers with mutant *RAS/RAF* are therapy refractory. Deregulated mRNA translation has become an emerging target in cancer treatment. We recently reported that mTOR inhibitors induce apoptosis via ER stress and the extrinsic pathway upon acute inhibition of the eIF4F complex in colon cancer cells and xenografts, while mutant BRAF600E leads to therapeutic resistance via ERK-mediated Mcl-1 stabilization. In this study, we demonstrated that several other translation inhibitors also activate ER stress and the extrinsic apoptotic pathway. Co-targeting translation and proteasome using the combination of Episilvestrol and Bortezomib promoted strong ER stress and rapid killing of colon cancer cells with mutant *RAS/RAF* in culture and mice. This combination led to marked induction of ER stress and ATF4/CHOP, followed by DR5- and BAX-dependent apoptosis, but unexpectedly with maintained or even increased levels of prosurvival factors such as p-AKT, p-4E-BP1, Mcl-1, and eiF4E targets c-Myc and Bcl-xL. Our study supports that targeting deregulated proteostasis is a promising approach for treating advanced colon cancer via induction of destructive ER stress that overcomes multiple resistance mechanisms associated with translation inhibition.

## INTRODUCTION

Colorectal cancer (CRC) represents the third leading cause of cancer death in the US and worldwide [1], and its development is driven by a series of genetic alternations. Tumor suppressor *APC* is lost in 85% of CRCs, leading to β-catenin stabilization, activation of Wnt targets such as c-Myc (Myc) and cyclin D1 and cancer initiation, which cooperates with hyperactivated RAS/RAF/ERK, and PI3K/AKT/mTOR signaling in cancer progression [2, 3]. Despite intense research and development of novel targeted therapies, the survival rates of colon cancer patients have improved only modestly. In particular, advanced CRCs with mutant K*RAS/BRAF* are resistant to targeted therapies such as EGFR and VEGF antibodies [4, 5]. Novel treatment strategies are therefore needed for these patients.

Translation is essential for normal development, and deregulated by oncogenes such as Myc, RAS, PI3K that lead to tissue- and cell-type specific production of oncogenic targets [6, 7] to enhance proliferation, survival, metastasis and other cancer phenotypes [8]. Translation initiation is considered the rate limiting step in protein biosynthesis, and regulated by the cap-binding protein complex eukaryotic initiation factor 4F (eIF4F) [6, 7, 9]. eIF4F comprises three major subunits: eIF4E, a cap-binding protein; eIF4A, an RNA helicase; and eIF4G, a scaffolding protein. eIF4A is the catalytic component, and belongs to the DEAD/DEHX box family of helicases that is involved in unwinding of the secondary structure of mRNA at the 5′-end, and thus facilitates the 43S preinitiation complex (PIC) scanning and recognition of the initiation codon and subsequent 60 S recruitment [6]. A variety of agents have been developed to target different steps in translation initiation, such as eIF4E, 4A, the binding of 4E/4G [6, 7], and upstream kinases including mTOR and ERK. Silvestrol and Episilvestrol are derivatives of rocaglate initially isolated from plants of *aglaia* genus. These compounds bind to eIF4A and impair RNA-dependent helicase function, and display potent anticancer activities in preclinical animal models with IC50 values in low nM ranges in culture [10].

The potency of translation targeting agents varies a great deal, while the underlying mechanisms remain unclear [6, 7]. Induction of apoptosis is a common therapeutic mechanism of anticancer agents [11], and regulated by the intrinsic or mitochondrial pathway [12, 13], and extrinsic or death receptor pathway [14]. Our recent work demonstrates that therapeutic effects of both allosteric and ATP-competitive mTOR inhibitors (mTORi) are mediated through induction of endoplasmic reticulum (ER stress) and apoptosis upon acute inhibition of eiF4E in colon cancer cells in culture and in mice, not the reversible growth suppression or inhibition on S6K induced by much lower doses [15]. *BRAFV600E* leads to therapeutic resistance through ERK-mediated Mcl-1 stabilization, which blocks Bid-mediated crosstalk with the mitochondrial pathway [16]. Silvestrol can induce G2/M arrest [17] or mitochondrial apoptosis [18].

The endoplasmic reticulum (ER) is central for the folding, maturation and secretion of newly synthesized proteins. Cancer cells can have increased ER stress due to deregulated translation and increased load on protein quality control including protein folding and degradation of misfolded proteins [19, 20]. PERK-mediated phosphorylation eIF2a (S51, p-eIF2a) is a major regulator of ER stress, which decreases general translation by inhibiting 43S PIC [6], but increases translation of ATF4 and its targets such as CHOP, GADD34 and chaperons in an adaptive response attempted for cell survival. However, prolonged ER stress leads to apoptosis through CHOP-dependent or -independent induction of DR5 and BH3-only proteins such as PUMA, Noxa and Bim [21, 22]. The ubiquitin (Ub)-proteasome system is the major protein degradation pathway and participates in ER-assisted degradation (ERAD) that removes unfolded and misfolded proteins. Inhibition of proteasome results in unfold protein response (UPR) or ER stress and cell death [23]. Bortezomib is an FDA-approved proteasome inhibitor [23, 24] and can induce apoptosis in a variety of cancer cells including CRC cells [25]. A variety of mechanisms have been proposed such as diminished prosurvival NF-κB signaling [23], activation of tumor suppressor p53 [25], and cytoplasmic release of Ca2+ [26].

In this study, we demonstrate that induction of proapoptotic ER stress is a key anticancer mechanism of eiF4F targeting agents. In particular, the combination of Episilvestrol and Bortezomib potentiates the killing of *RAS/RAF* mutated colon cancer cells via destructive ER stress and activation of both the extrinsic and intrinsic apoptotic pathways. These results suggest that targeting deregulated proteostasis might be an effective strategy for treating advanced colon cancer.

## RESULTS

### Translation inhibitors induce ER stress and the death receptor pathway in colon cancer cells

**Figure 1:**
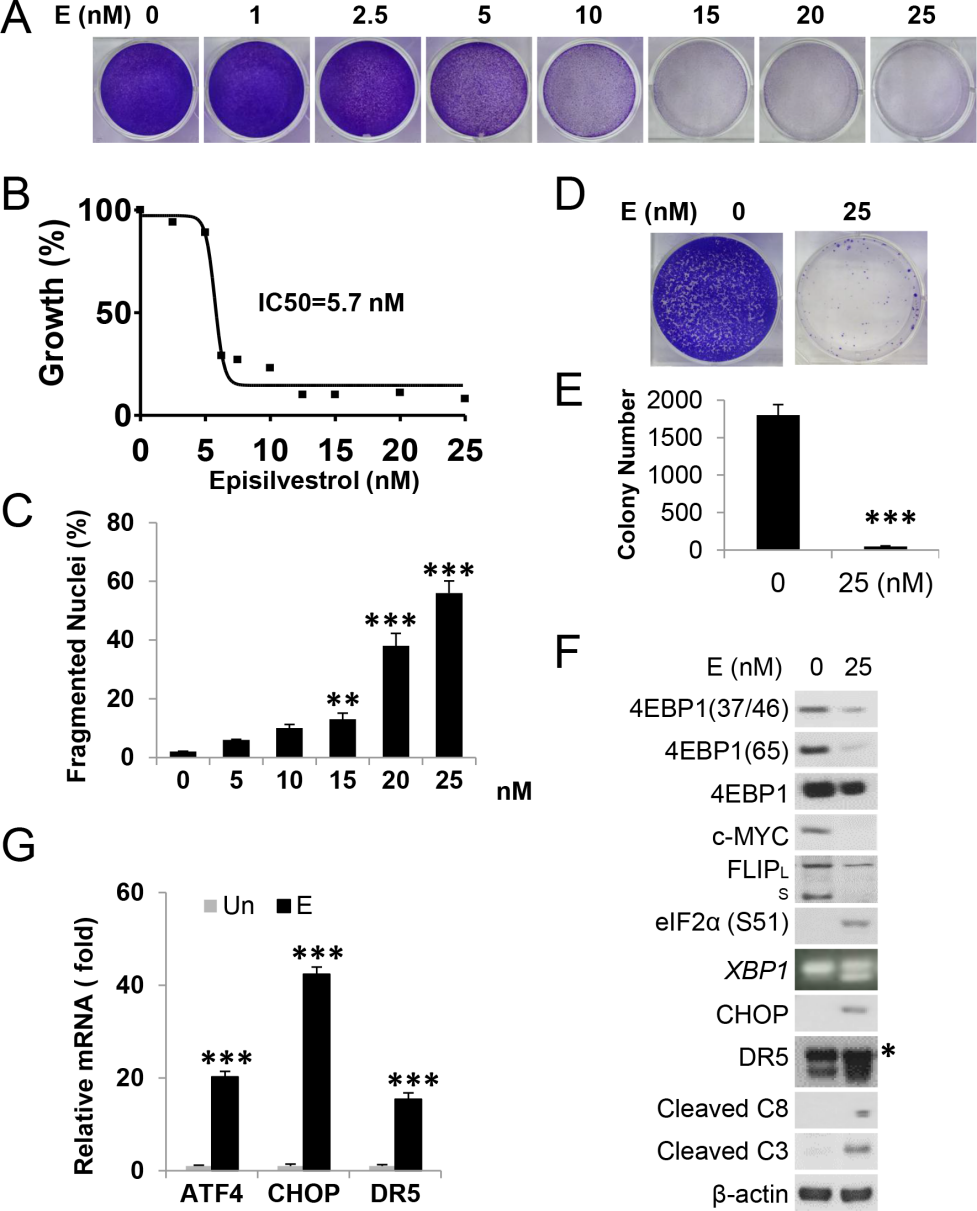
Episilvestrol activates ER stress and apoptosis in HCT 116 cells HCT 116 cells were treated with Episilvestrol. (**A**) Adherent cells were stained by crystal violet 48 h after treatment with increasing doses of Episilvestrol. (**B**) Cell proliferation was measured by MTS assay in cells treated as in A. IC50 value was calculated using Prism VII. (**C**) Quantification of condensed and fragmented nuclei 48 h after treatment. (**D**) Colony formation of cells after 24 h treatment, followed by growth in drug-free medium for 14 days before crystal violet staining. Representative pictures are shown. (**E**) Quantification of colonies in D. (**F**) Indicated proteins were analyzed in cells 24 h after treatment by western blotting. C3, caspase-3; C8, caspase-8. * Non-specific upper band (DR5). FLIP_L/S_ are 56 and 28 KD respectively. β-actin was used as a loading control. *XBP1* was analyzed by RT-PCR. (**G**) mRNA levels of *ATF4*, *CHOP* and *DR5* in cells treated as in F were analyzed by real-time PCR. The levels in vehicle (Un) treated cells were set at 1. C, E and G, values represent mean + s.d. (*n* = 3). **P < 0.01, ***P < 0.001, E *vs*. Un (Student's *t*-test, two tailed).

We first determined the effects of Episilvestrol as a single agent on the growth of human colon cancer cell HCT 116 (*KRASG13D*). Episilvestrol caused dose-dependent growth suppression at nM concentrations within 48 hours (Figure 1A and Supplementary Figure S1A), with a half maximal inhibitory concentration value (IC50) of 5.7 nM (Figure 1B). Episilvestrol also induced dose-dependent nuclear fragmentation (Figure 1C), a hallmark of apoptosis, and diminished long-term survival following a 24 hour exposure (25 nM) (Figure 1D and 1E). We then analyzed a set of biochemical markers on translation, ER stress and apoptosis 24 hours after treatment. We detected a strong reduction in p-4E-BP1 (37/46, 65), and survival factors such as c-Myc, c-FLIP (_L/S_), Mcl-1, p-AKT and p-FoxO1/3 [27] (Figure 1F and Supplementary Figure S1B). The reduction in c-FLIP and Myc might be due to impaired eiF4E-dependent translation, or increased cleavage or degradation. On the other hand, a strong induction of ER stress and apoptosis markers was observed, including p-eiF2a, CHOP and target DR5, spliced *XBP1*, cleaved caspase-3 (C3), caspase-8 (C8), with diminished levels in the ER chaperon Bip/GRP-78 (Figure 1F, Supplementary Figure S1B). Induction of *ATF4*, *CHOP*, and *DR5* transcripts was confirmed by reverse transcription PCR (RT-PCR) analysis (Figure 1G).

We then studied a second translation inhibitor 4EGI-1, which prevents the binding of eiF4E and eiF4G [7]. 4EGI-1 treatment induced dose-dependent growth suppression and apoptosis in HCT116 cells (Supplementary Figure S2A–S2D) associated with inhibition of mTOR signaling and induction of ER stress. Significant loss in p-4E-BP1 (37/46) and p-AKT (S473), and induction *CHOP*, *DR5*, and *XBP1 splicing* was correlated with caspase-3 activation and apoptosis (Supplementary Figure S2E and S2F). The IC50 of 4EGI-1 is 22 uM (Supplementary Figure S2B), comparable to mTORi Everolimus (∼15 uM) [15], over 3000 thousand fold higher than Episilvestrol (5.7 nM). These results indicate that structurally and functionally diverse translation inhibitors activate ER stress and the death receptor apoptotic pathway in colon cancer cells.

### Episilvestrol induces ER stress and apoptosis in colon cancer cell lines

**Figure 2:**
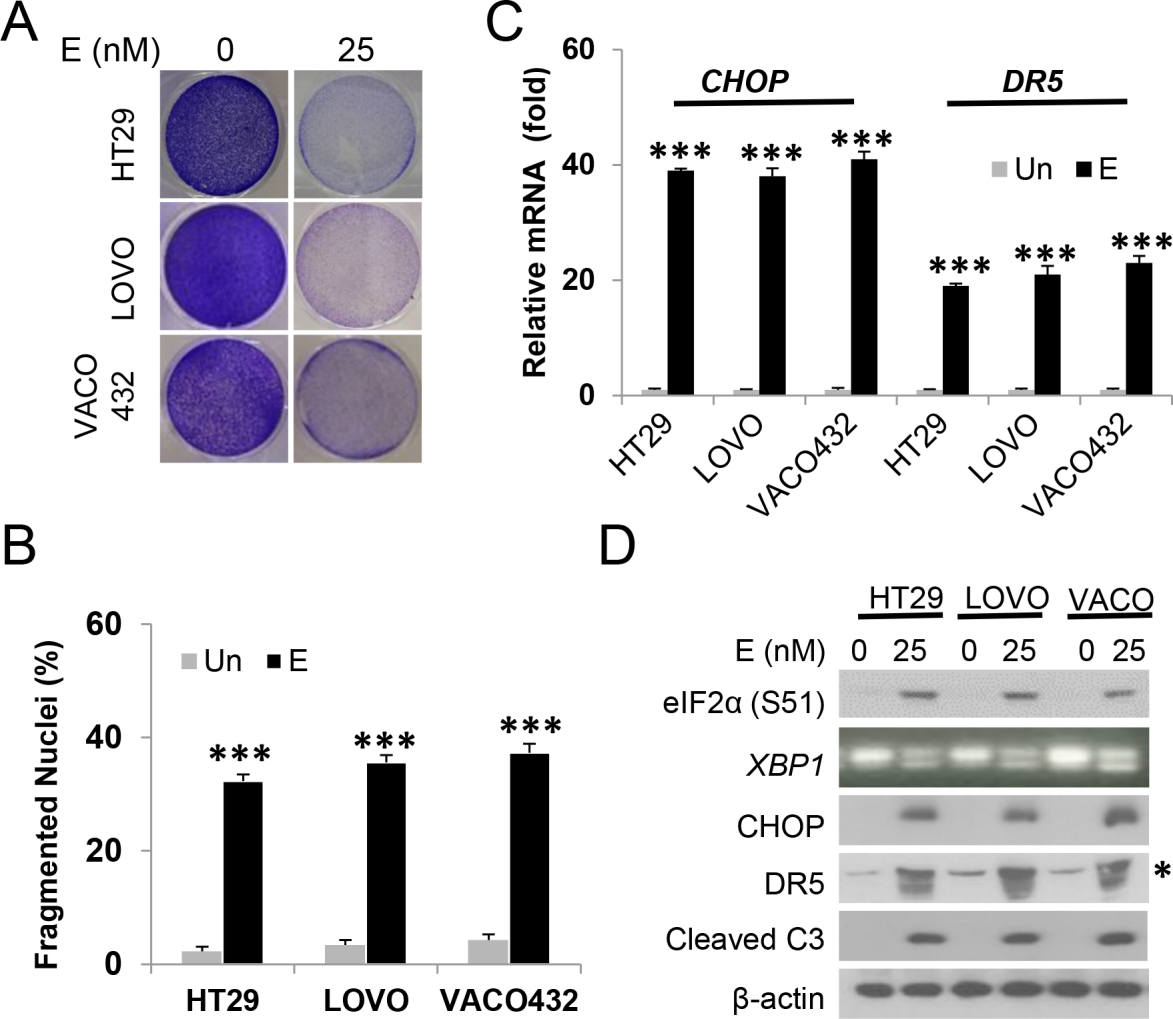
Episilvestrol induces ER stress and apoptosis in colon cancer cells HT29, LOVO and VACO432 cells were treated with 25 nM Episilvestrol. (**A**) Adherent cells were stained by crystal violet 48 h after treatment. (**B**) Quantification of condensed and fragmented nuclei in cells treated as in A. (**C**) mRNA levels of *CHOP* and *DR5* in cells 24 h after treatment were analyzed by real-time PCR. The levels in vehicle (Un) treated cells were set at 1. (**D**) Indicated proteins in cells 24 h after treatment were analyzed by western blotting. *Non-specific upper band (DR5). *XBP1* was analyzed by RT-PCR. B, C, values represent mean + s.d. (*n* = 3). ***P < 0.001, E *vs*. Un (Student's *t*-test, two tailed).

To further investigate the effects of Episilvestrol in *KRAS/BRAF* mutated colon cancer, we selected four more cell lines, HT29 (*BRAF600E*), VACO432 (*BRAF600E*), LOVO (*KRASG13D* and SW480 (*KRASG12V*). These lines have diverse genetic backgrounds, differing in mutational status in *APC*, β-catenin, *PIK3CA*, *p53*, and mismatch repair genes [16, 28]. Episilvestrol induced growth suppression and apoptosis at 25 nM in all these lines (Figure 2A and 2B and data not shown). Apoptosis was correlated with the induction of *CHOP* and *DR5* transcripts and protein, and elevated p-eIF2α and caspase-3 cleavage (Figure 2C and 2D). The above data support that targeting translation initiation via eiF4F or its negative regulator 4E-BP1 [15, 16] induces ER stress and apoptosis in colon cancer cells, while the 4A targeting agent Episilvestrol is the most potent.

### The Episilvestrol and Bortezomib combination potentiates apoptosis in colon cancer cells

**Figure 3:**
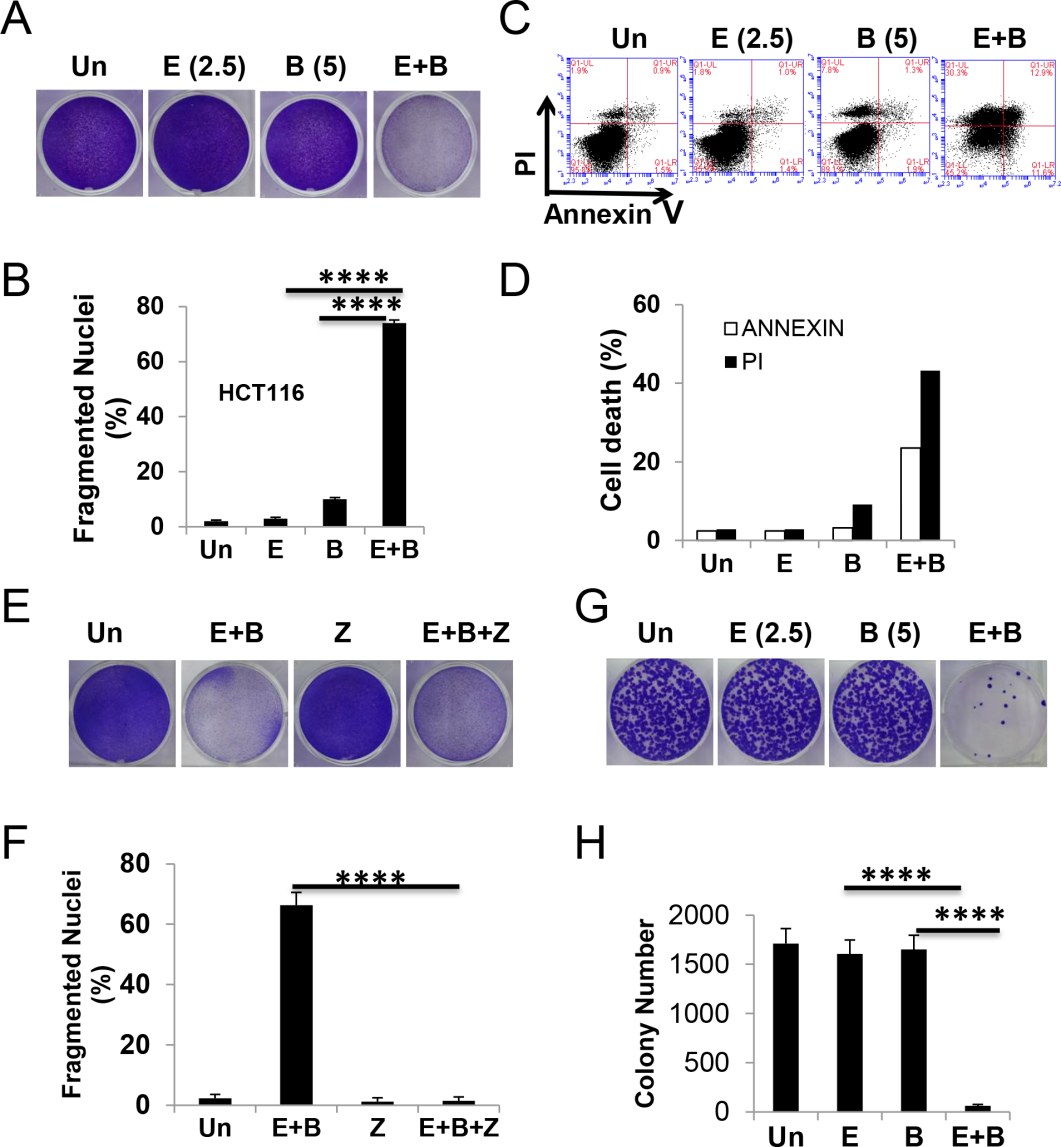
The Episilvestrol and Bortezomib combination potentiates the killing of HCT 116 cells HCT 116 cells were treated with vehicle (Un), Episilvestrol (E, 2.5 nM), Bortezomib (B, 5 nM), or their combination (E+B). (**A**) Adherent cells were stained by crystal violet 48 h after treatment. (**B**) Quantification of condensed and fragmented nuclei in cells treated as in A. (**C**) Cells were treated as in A, stained with Annexin V/propidium iodide, and analyzed by flow cytometry. (**D**) Quantitation of Annexin V+ and PI + cells in C. (**E**) Adherent cells were stained by crystal violet 48 h after treatment with or without z-VAD (Z, 20 μM). (**F**) Quantification of condensed and fragmented nuclei in cells treated as in E. (**G**) Colony formation of cells after 24 h treatment, followed by growth in drug free medium for 14 days before crystal violet staining. Representative pictures are shown. (**H**) Quantification of colonies in E. B, F and H, values represent mean + s.d (*n* = 3). ****P < 0.0001, E+B *vs*., E, B, or E+B+Z (Multiple comparisons by one way ANOVA followed by Turkey Test).

We reasoned that potentiating ER stress through drug combination might enhance cancer cell killing while reducing normal tissue toxicity. Through a small screen of known ER stressors, we found that the combination of proteasome inhibitor Bortezomib (5 nM) with a non-toxic dose of Episilvestrol (2.5 nM) induces greater than additive growth suppression in HCT 116 cells (Figure 3A and Supplementary Figure S3A). In contrast, little or no additive effects were found with 5-FU, the corner stone of CRC chemotherapy, kinase inhibitor Regorafenib, Hsp90 inhibitor 17-DMAG, or mTOR inhibitor Everolimus (Rad001) (data not shown). The Episilvestrol and Bortezomib combination resulted in extensive apoptosis as measured by nuclear fragmentation and flow cytometry (Figure 3B, and 3D, and Supplementary Figure S3B), which was blocked by the pan-caspase inhibitor z-VAD (Figure 3E and 3F). A transient 24-hour exposure to the combination diminished long-term clonogenic survival (Figure 3G and 3H). These doses were significantly below respective IC50s of single agent, 5.7 nM and 10.8 nM (Figure 1B and Supplementary Figure S3C), and caused little or no toxicity or apoptotic induction (Figure 3).

### The Episilvestrol and Bortezomib combination activates ER stress-dependent apoptosis in colon cancer cells

**Figure 4:**
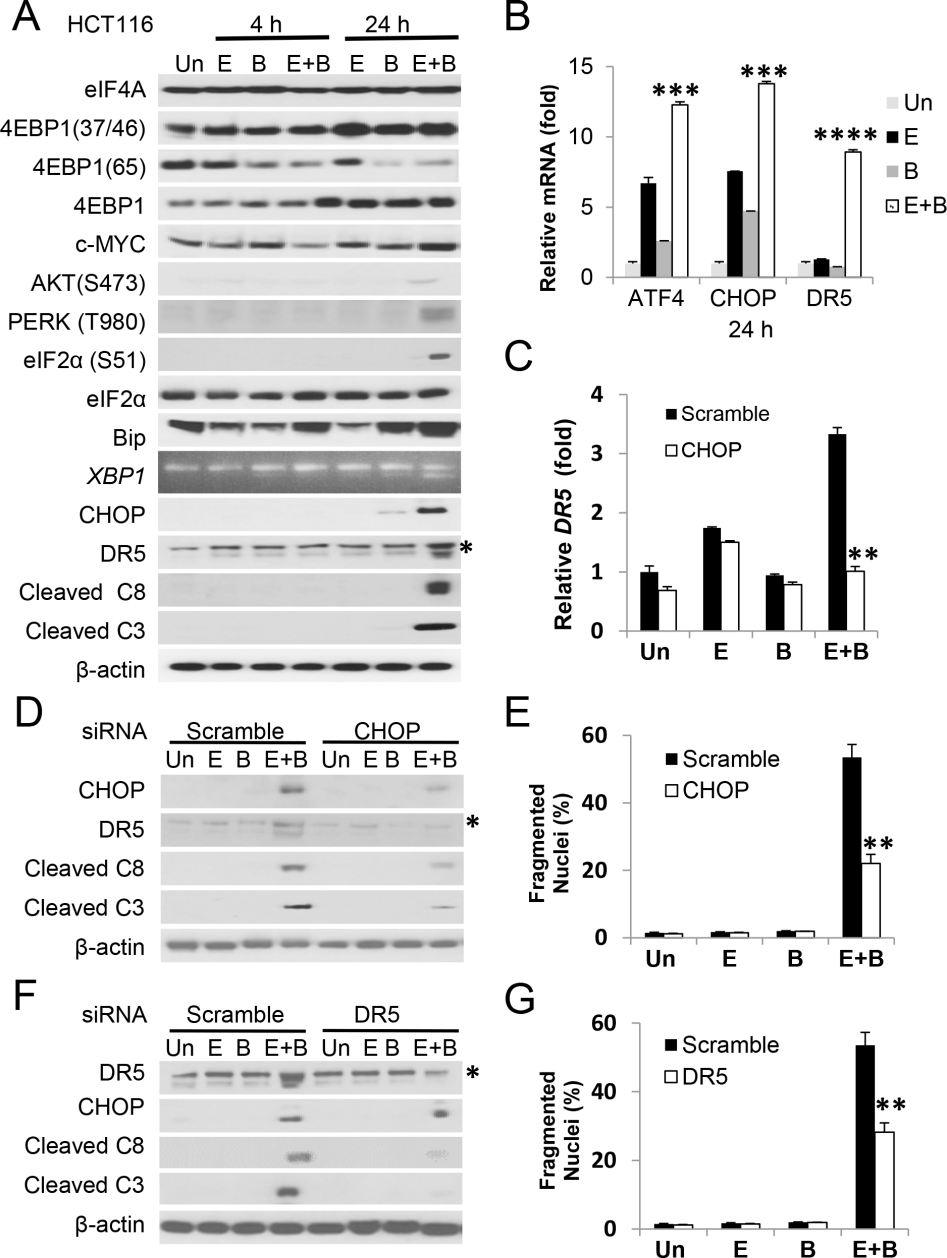
The Episilvestrol and Bortezomib combination induces ER stress-dependent apoptosis in HCT 116 cells HCT 116 cells were treated with vehicle (Un), Episilvestrol (E, 2.5 nM), Bortezomib (B, 5 nM), or their combination (E+B). (**A**) Indicated proteins in cells 4 h and 24 h after treatment were analyzed by western blotting. *Non-specific upper band (DR5). *XBP1* was analyzed by RT-PCR. (**B**) The indicated mRNAs were analyzed by real-time PCR. The levels in vehicle (Un) treated cells were set at 1. (**C**) Cells were transfected with either scramble or *CHOP* siRNA 24 h prior to drug treatment. *DR5* mRNA was analyzed 24 h after drug treatment by RT–PCR. (**D**) The indicated proteins were analyzed in cells 24 h after drug treatment by western blotting. (**E**) Quantification of condensed and fragmented nuclei in cells 48 h after treatment. (**F**) Cells were transfected with either scramble or *CHOP* siRNA 24 h prior to drug treatment. The indicated proteins were analyzed as in D. (**G**) Quantification of condensed and fragmented nuclei was analyzed as in E. B, C, E and G, values represent mean + s.d. (*n* = 3). ***P < 0.001, ****P < 0.0001, E+B *vs*. E or B (B, Multiple comparisons by one way ANOVA followed by Turkey Test), and scramble *vs*. siRNA (Student's *t*-test, two tailed).

To understand the basis of rapid apoptosis induced by Episilvestrol and Bortezomib combination, we first examined the ER stress and translation signaling pathways. We found that the combination treatment strongly elevated p-eIF2a, CHOP, Bip, *XBP1* splicing, and apoptosis effectors such as DR5, cleaved caspase-3 and -8 within 24 hours, but surprisingly little or no decrease in p-4EBP1 (S37/46), p-AKT (473), or eiF4E targets c-Myc, Bcl-xL [6, 7], or Mcl-1 [15] (Figure 4A and Supplementary Figure S4A), previously described resistance mechanisms of translation inhibitors. Enhanced transcriptional activation by the combination in *ATF4*, *CHOP*, *GADD34*, *DR5* and *TNFR1*, but not the DR5 ligand *TRAIL*, was confirmed by RT-PCR (Figure 4B and Supplementary Figure S4B). Using siRNA, we demonstrated that induction of DR5 transcript and protein, caspase cleavage and apoptosis was strongly inhibited by *CHOP* knockdown (Figure 4C, and 4E). Apoptosis, but not CHOP induction, was also significantly reduced by *DR5* siRNA (Figure 4F and 4G).

**Figure 5:**
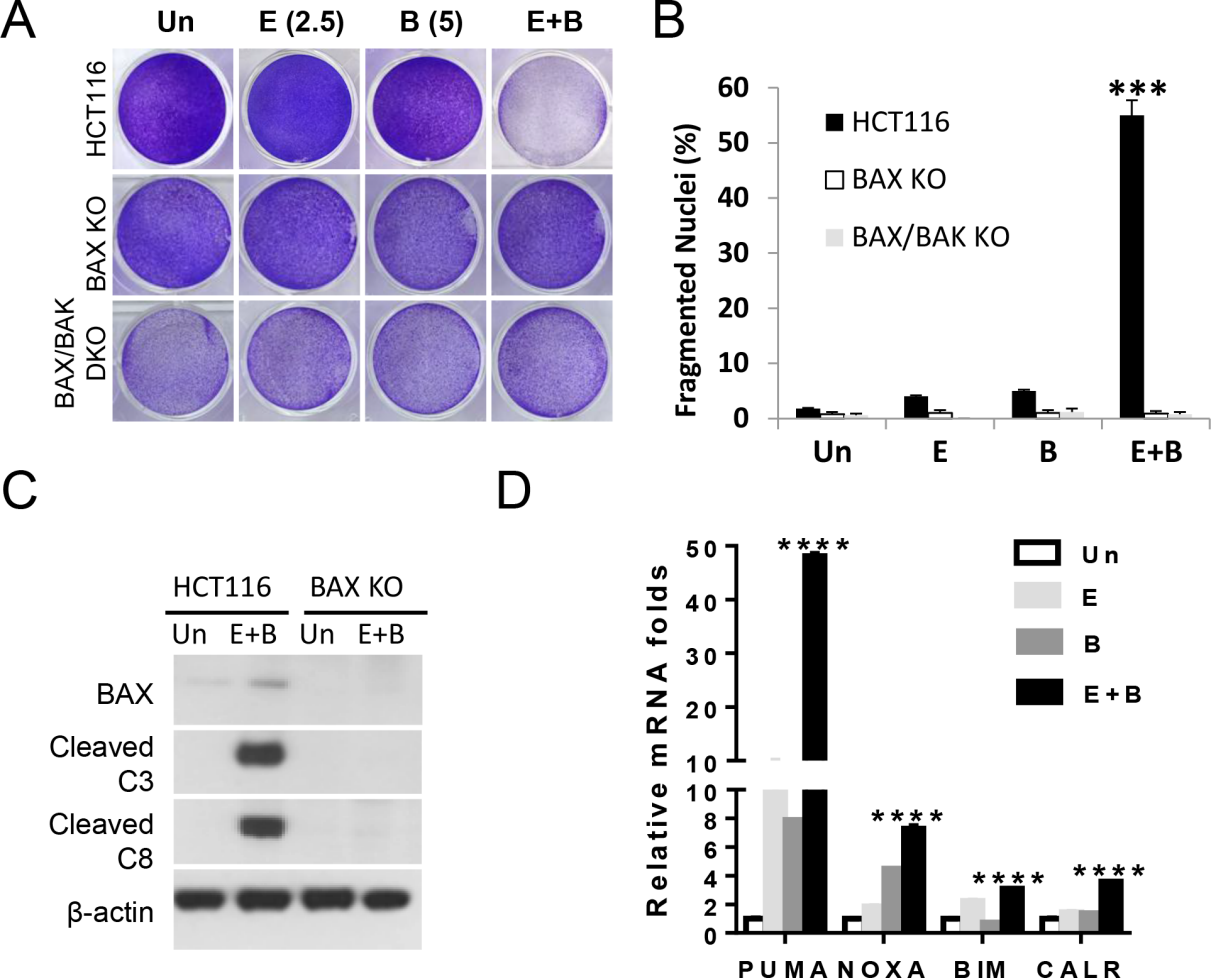
The Episilvestrol and Bortezomib combination activates the mitochondrial apoptotic pathway in HCT 116 cells HCT 116 cells, isogenic *BAX* KO or *BAX/BAK* DKO cells were treated with vehicle (Un), Episilvestrol (E, 2.5 nM), Bortezomib (B, 5 nM), or their combination (E+B). (**A**) Adherent cells were stained by crystal violet 48 h after treatment. (**B**) Quantification of condensed and fragmented nuclei in cells treated as in A. (**C**) Indicated proteins in 24 h after treatment were analyzed by western blotting. (**D**) The indicated mRNAs were analyzed by real-time PCR. The levels in vehicle (Un) treated cells were set at 1. B, and D, values represent mean+s.d. (*n* = 3). ***P < 0.001, WT *vs*. KO (B, Student's *t*-test, two tailed), ****P < 0.0001, E+B *vs*. E or B (D, Multiple comparisons by one way ANOVA followed by Turkey Test).

The BH3-only protein Bid was cleaved in the combination group (Supplementary Figure S4A), suggesting amplification of apoptotic signal through the mitochondrial pathway. In addition, HCT 116 cells deficient in *BAX* (*BAX* KO) or *BAX and BAK (BAX/BAK* DKO) were highly resistant to growth suppression, apoptosis and cleavage of capspase-8 and caspase-3 induced by the combination (Figure 5A, and 5C). These data are consistent with Bax as the major apoptotic mediator in HCT 116 cells in response to Bortezomib and other anticancer agents [4, 29], and capspase-3 can regulate caspase-8 cleavage in a FADD-containing complex [30]. ER stress is known to transcriptionally activate BH3-only proteins and immune modulators [21, 22]. The combination treatment resulted in marked induction of *PUMA* (48-fold), and at lesser extent *Noxa* (7.2-fold), *Bim* (3-fold) and *Calreticulin* (*CALR)* (3.5-fold) (Figure 5D).

### The Episilvestrol and Bortezomib combination potently kills *KRAS/BRAF* mutant colon cancer cells

**Figure 6:**
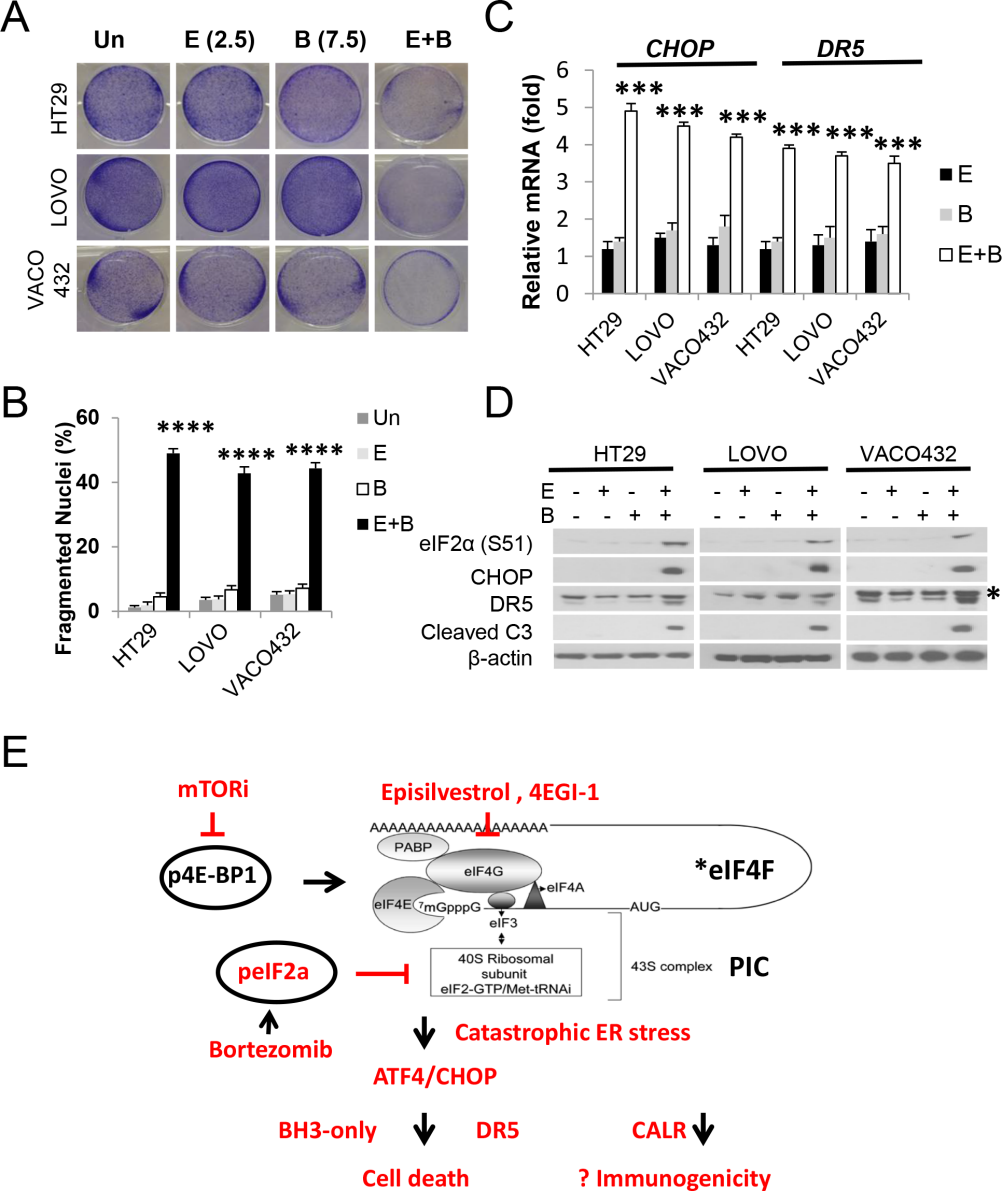
Episilvestrol and Bortezomib combination potently killings KRAS/BRAF mutant colon cancer cells HT29, LOVO and VACO432 cells were treated with vehicle (Un), Episilvestrol (E, 2.5 nM), Bortezomib (B, 7.5 nM), or their combination (E+B). (**A**) Adherent cells were stained by crystal violet 48 h after treatment. (**B**) Quantification of condensed and fragmented nuclei in cells treated as in A. (**C**) mRNA levels of *CHO*P and *DR5* in cells 24 h after treatment were analyzed by real-time PCR. The levels in vehicle (Un) treated cells were set at 1. (**D**) Indicated proteins in cells treated as in C were analyzed by western blotting. B and C, values represent mean + s.d. (*n* = 3). ***P < 0.001, ****P < 0.0001, E+B *vs*. E or B (Multiple comparisons by one way ANOVA followed by Turkey Test). (**E**) Proposed model. Mutational activation of Wnt and RAS/RAF deregulates *eiF4F in colon cancer cells. Dual inhibition of translation initiation via eiF4F and 43S PIC leads to hyperactivation of p-eiF2a/ATF4/CHOP, catastrophic ER stress, and rapid cell death with increased immunogenicity.

To extend our findings, we subjected six additional *KRAS/BRAF* mutant CRC cell lines to Episilvestrol and Bortezomib combination, or single agent. The combination strongly suppressed the growth of all cell lines (Figure 6A and Supplementary Figure S5), which was associated with rapid apoptosis, caspase-3 cleavage and ER stress (p-eiF2a, CHOP and DR5) (Figure 6B–6D). The above findings clearly demonstrate that co-targeting translation and proteasome leads to rapid killing of CRC cells via destructive ER stress and ATF4/CHOP hyperactivation, engaging both the extrinsic and intrinsic pathways to override multiple well-described resistant mechanisms associated with the use of translation inhibitors. The dual inhibition of eiF4F and eiF2a, two key steps in translation initiation, likely potentiates ER stress, cell death and immunogenicity (Figure 6E).

### Induction of ER stress and apoptosis mediates *in vivo* antitumor activities of Episilvestrol and Bortezomib combination

**Figure 7:**
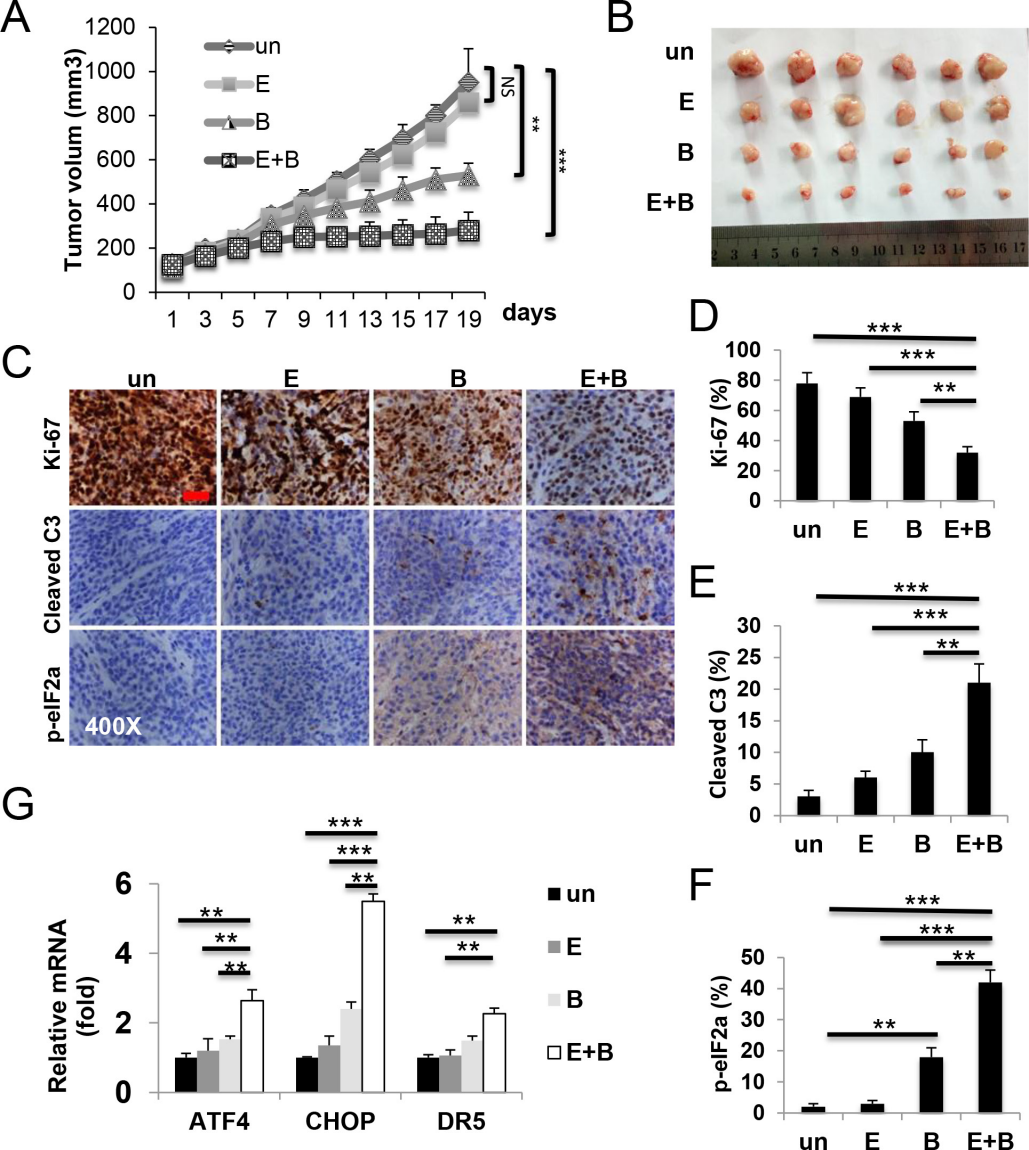
Episilvestrol and Bortezomib combination induces ER stress and apoptosis in xenograft tumors Tumor bearing nude mice were randomized and treated with vehicle, 0.5 mg/kg Episilvestrol, 0.5 mg/kg Bortezomib, or their combination 3 times a week for total of 10 times. (**A**) Tumor volume was plotted. *N* = 7 tumors/group. *P < 0.05 (Student's *t*-test, two-tailed). (**B**) Representative images of tumors at the end of experiments. (**C**) Representative images of Ki-67, cleaved caspase-3 and p-eIF2a staining in tumors, and (**D**–**F**) quantitation 24 h after the 3^rd^ treatment Scale bar: 100 μM, original magnification 400×. (**G**) The indicated mRNAs in 3 randomly chosen tumors/group were analyzed by RT-PCR. The levels in vehicle (Un) treated cells were set at 1. Values represent mean + s.d. (*n* = 3). **P < 0.01, ***P < 0.001 (Multiple comparisons by one way ANOVA followed by Turkey Test).

To examine anti-tumor activities of Episilvestrol and Bortezomib combination *in vivo* and underlying mechanisms, we randomized tumor bearing mice to receive three different doses of single agent and combination. At the lowest dose of 0.25 mg/kg/day, three days a week, little or no weight loss or tumor response was observed in single agent or combination group after 10 treatments (Supplementary Figure S6A and data not shown). At 0.5 mg/kg, the combination group displayed potent inhibition of tumor growth showed little or no weight loss (Supplementary Figure S6B). Episilvestrol or Bortezomib alone group showed limited or modest tumor response [31] (Figure 7A and 7B). Histological and molecular analysis on tumors harvested after three treatments revealed marked inhibition of cell proliferation evident by Ki-67 staining, and induction of ER stress and apoptosis evident by p-eIF2a and cleaved caspase-3 staining, and elevated *CHOP* and *DR5*, in the combination group, significantly increased from single agent groups (Figure 7C–7G). However, at 1 mg/kg, Bortezomib alone, or in combination with Episilvestrol resulted in significant mortality. Episilvestrol treatment alone did not result in weight loss even at the highest dose (Supplementary Figure S6C). These data demonstrate that induction of ER stress and apoptosis contributes to *in vivo* antitumor activities of Episilvestrol and Bortezomib combination.

## DISCUSSION

Despite intense research and the availability of FDA-approved targeting agents, advanced CRCs remain challenging. Targeting individual drivers in CRCs has limited success due to lack of agents against the gatekeeper *APC* mutation or Wnt/Myc hyperactivation, and mutant *KRAS/BRAF*-mediated poor prognosis or resistance to targeted therapies [3, 32]. In this study, we discovered that different classes of translation inhibitors kill CRC cells via ER stress and activation of the death receptor pathway. The combination of the eiF4A inhibitor Episilvestrol and Bortezomib potentiates the killing of a wide variety of colon cancer cells with mutant *KRAS/BRAF*. The cell death and caspase activation is preceded by strong elevation in p-eIF2a and ATF4/CHOP, and dependent on DR5 and BAX. Single agent at the same dose has limited toxicity or ability to induce ER stress. These data demonstrate that Episilvestrol and Bortezomib combination targets deregulated proteostasis in therapy-refractory CRCs.

Translation initiation is an essential cellular process and highly regulated by the availability of nutrients, growth factors, and cellular energy levels [33]. Deregulated translation is caused by numerous oncogenes and leads to increased proliferation, survival, invasion and altered metabolism. However, eiF4E-dependent translation of oncogenic targets is thought to be tissue and cell-type specific [6, 7]. Therefore, targeting translation initiation in cancer remains challenging due to its physiology role, cell-type specific targets, and a lack of clear mechanistic understanding of existing agents [6, 7]. The current study demonstrates that the potency of translation inhibitors in CRC cells are closely associated by their ability to induce ER stress, hyperactivation of ATF4/CHOP and apoptosis. Potentiation of the TRAIL-induced apoptosis by 4EGI-1 in lung cancer cells supports the involvement of the extrinsic pathway [34]. It would be interesting to determine if this mechanism is applicable to agents targeting translation via cap-binding, *i.e*. 4EI-1 and or upstream kinases such as Mnk1/2, ERK, and modulated by mutant *RAS/RAF*.

The efficacy of translation inhibitor [6, 7] or ER stress inducer (Supplementary Figure S6) [23] monotherapy is likely to be limited in solid tumors due to toxicity as well as intrinsic and acquired resistance. Resistance mechanisms of mTORi, including FDA-approved rapalogs, are very complex, ranging from activation of PI3K/AKT [35], ERK [36], incomplete inhibition on p 4E-BP1 [37], or mutant *BRAF/KRAS* or *APC* reported recently by us [15, 16]. Cap-independent c-Myc translation [38] and overexpression of the ABCB1/P-glycoprotein was linked to silvestrol resistance [39, 40]. Combination therapies offer potential benefits for inhibiting multiple targets and signaling pathways to effectively kill cancer cells and preventing/delaying the emergence of drug resistance [4, 5]. The Episilvestrol and Bortezomib combination potently induces CRC cell killing with very little or no reduction in well-established eiF4E oncotargets such as c-Myc, Bcl-xL or its regulators such as p-4E-BP1 (37/46) or p-AKT [6, 7], distinct from that induced by single agent at much higher doses. These findings suggest that this combination targets deregulated translation/protein degradation in cancer cells, rather than a few eiF4E oncogenic targets *per se*, to trigger rapid activation of apoptosis and help prevent or delay the development of resistance. Significant dose reduction in the setting of combination likely decreases normal tissue toxicity and long-term complications in cancer patients and survivors.

Cancer cells often show elevated rates of protein synthesis and ER stress [19, 20], which can be pro-survival and pro-death [22, 41, 42]. Our work suggests an interesting possibility that deregulated proteostasis with overstressed protein quality control is a druggable vulnerability of colon cancers driven by Wnt/Myc and mutant *RAS/RAF* [3], which predisposes to hyperactivation of ATF4/CHOP and catastrophic ER stress. The dual inhibition of eiF4F and eiF2a by the Episilvestrol and Bortezomib combination likely potentiates this response (Figure 6E). The induction of p-eiF2a and release of damage associated molecular patterns (DAMPs) including Calreticulin (CALR) might be a biomarker for immunogenic cell death, capable of activating cytotoxic T cells (CTLs) and long-term immune memory as suggested [43]. This might be particularly relevant for *in vivo* therapeutic efficacy, given that modes of cell death [4, 43, 44], and mutational load [45] can strongly impact the immune response. For examples, *FADD or Caspase-8* KO leads to lethal activation of RIP1/RIP3-dependent necroptosis and inflammation in mice [46, 47], but well-tolerated in CRC cells [15] with reduced or lost expression of RIP3 [30]. It will therefore be interesting to determine if p-eiF2a modulates therapeutic responses via engaging the immune system in future studies.

In summary, our preclinical data demonstrate that induction of pro-apoptotic ER stress is an important antitumor mechanism of translation inhibitors. Co-targeting translation and proteasome potentiates the killing of colon cancer cells with mutant *RAS/RAF* via catastrophic ER stress, and rapid activation of both the extrinsic and intrinsic apoptosis to overcome multiple resistance mechanisms. Our study provides a strong rationale and potential biomarkers for future testing of such combinations in advanced and therapy refractory CRCs through both cell-intrinsic and potential crosstalk with the immune system.

## MATERIALS AND METHODS

### Cell culture and treatment

The human CRC cell lines, including HCT116 (*KRASG13D*), LOVO (*KRASG13D*), DLD1 (*KRASG13D*), SW480 (*KRASG12V*), HT29 (*BRAFV600E*), VACO432 (*BRAFV600E*), and RKO (*BRAFV600E*) were obtained from the American Type Culture Collection (Manassas, VA, USA). The detailed genetic characteristics can be found in the Cancer Genome Project tumor cell line database (http://www.sanger.ac.uk/genetics/CGP/). Isogenic HCT 116 *BAX* knockout (KO) [29] cells were from Bert Vogelstein, and *BAX/BAK* double KO (DKO) cells [48] were from Richard J. Youle. Cell lines were last tested for the absence of Mycoplasma, genotype, drug response and morphology in our laboratory in August 2016. We examined loss of expression of targeted proteins by western blotting routinely; no additional authentication was done by the authors. Details on cell culture and chemicals are found in the supplementary materials.

### Western blotting

Western blotting was performed as previously described [49]. Details on antibodies are found in the supplementary materials.

### Real-time PCR

Total RNA was isolated from cells using the Mini RNA Isolation II Kit (Zymo Research, Orange, CA, USA) according to the manufacturer's protocol. One microgram of the total RNA was used to generate complementary DNA using Superscript II reverse transcriptase (Invitrogen, Carlsbad, CA, USA) [50]. Real-time PCR was carried out as described [15]. Details on primers are found in the Supplementary Materials (Supplementary Table S1).

### Analysis of cell viability, apoptosis and cell death

Cell growth was measured by MTS, and apoptosis was analyzed by nuclear staining with Hoechst 33258 (Invitrogen) and Annexin V/propidium iodide (Invitrogen) staining followed by flow cytometry as described [15]. For crystal violet assays, the same number of cells were treated for 48 hours in 12-well plates, and the attached cells were stained with crystal violet (Sigma, St. Louis, MO) [49]. Details are found in the supplementary materials.

### Colony formation assay

Cells were treated and plated in 12-well plates at appropriate dilutions, and allowed to grow for 10–14 days before staining with crystal violet (Sigma, St. Louis, MO, USA) [15, 51].

### Transfection

Transfection was performed using Lipofectamine 2000 according to the manufacturer's instructions. Small-interfering RNA (siRNA) duplexes were synthesized by Dharmacon (Lafayette, CO, USA). Details for transfection, drug treatment and siRNA sequence are found in the supplementary materials.

### Xenograft studies

All animal experiments were approved by the University of Pittsburgh Institutional Animal Care and Use Committee. Female 5–6 week-old Nu/Nu mice (Charles River, Wilmington, MA) were housed in a sterile environment with micro isolator cages and allowed access to water and chow *ad libitum*. Mice were injected subcutaneously in both flanks with 4 × 10^6^ WT HCT116 cells. After implantation, tumors were allowed to grow to 100 mm^3^, approximately 10 days before treatment was initiated. Mice were randomized into four groups to receive either vehicle or Episilvestrol (0.25, 0.5 or 1 mg/kg) or Bortezomib (0.25, 0.5 or 1 mg/kg) or their combination in saline three times a week by intraperitoneal injection (I.P.) Detailed methods on tumor measurements and analysis are found in the Supplementary Materials as described [52, 53].

### Statistical analysis

GraphPad Prism VII software (La Jolla, CA, USA) was used for statistical analyses. Data was analyzed by unpaired Student's *t*-test (two-tailed) or analysis of variance (ANOVA) followed by Turkey Test in which multiple comparisons were performed using the method of least significant difference. *P* values were considered significant if *p* < 0.05. The means ± one standard deviation (s.d.) were displayed in the Figures.

## SUPPLEMENTARY MATERIALS FIGURES AND TABLES



## Abbreviations

ER: endoplasmic reticulum
CHOP: C/EBP homologous protein
DR5: Death receptor 5
FADD: Fas-associated death domain protein
