# Correction: Co-targeting translation and proteasome rapidly kills colon cancer cells with mutant *RAS/RAF* via ER stress

**DOI:** 10.18632/oncotarget.24288

**Published:** 2018-01-22

**Authors:** Xiangyun Li, Mei Li, Hang Ruan, Wei Qiu, Xiang Xu, Zhang Lin, Jian Yu

**Affiliations:** ^1^ First department, State Key Laboratory of Trauma, Burn and Combined Injury, Research Institute of Surgery and Daping Hospital, Third Military Medical University, Daping, Yu Zhong District, Chongqing 400042, P.R. China; ^2^ Department of Animal Genetics, Breeding and Reproduction, Nanjing Agricultural University, Weigang, Nanjing 210095, P.R. China; ^3^ Department of Pathology, University of Pittsburgh Cancer Institute, Pittsburgh, PA 15213, USA; ^4^ Department of Pharmacology and Chemical Biology, University of Pittsburgh School of Medicine, University of Pittsburgh Cancer Institute, Pittsburgh, PA 15213, USA

This article has been corrected: Dr. Mei Li is now listed as the first correspondence author instead of Dr. Jian Yu in the correspondence section.

Original article: Oncotarget. 2017; 8:9280-9292. https://doi.org/10.18632/oncotarget.14063

