# Supplementary material for: Co-targeting translation and proteasome rapidly kills colon cancer cells with mutant RAS/RAF via ER stress
Source: Oncotarget. 2016 Dec 21;8(6):9280–92. doi: 10.18632/oncotarget.14063 (PMC5354731; doi:10.18632/oncotarget.14063)
Supplement: Supplementary file 1 [file oncotarget-08-9280-s001.pdf]

Co-targeting translation and proteasome rapidly kills colon cancer cells with mutant *RAS/RAF* via ER stress

Supplementary Materials

Cell culture and treatment

The human CRC cell lines, including HCT116 (*KRASG13D*), LOVO (*KRASG13D*), DLD1 (*KRASG13D*), SW480 (*KRASG12V*), HT29 (*BRAFV600E*), VACO432 (*BRAFV600E*), and RKO (*BRAF600E*) were obtained from the American Type Culture Collection (Manassas, VA, USA). The detailed genetic characteristics can be found in the Cancer Genome Project tumor cell line database (http://www.sanger.ac.uk/genetics/CGP/). Isogenic HCT 116 *BAX* knockout (KO) cells [1] were from Bert Vogelstein, and *BAX/BAK* double KO (DKO) cells [2] were from Richard J. Youle. All cell lines were cultured in McCoy's 5A modified medium (Invitrogen, Carlsbad, CA, USA) supplemented with 10% defined fetal bovine serum (Hyclone, Logan, UT, USA), 100 units/mL penicillin, and 100 μg/mL streptomycin (Invitrogen). Cells were maintained in a 37°C incubator at 5% CO2. For drug treatment, cells were plated in 12-well plates at 20–30% density 24 h prior to treatment. The DMSO (Sigma, St Louis, MO, USA) stocks of the agents used: including Bortezomib (LC Laboratories, Woburn, MA, USA), Episilvestrol (Shanghai BetterBioChem Co., Ltd. Shanghai, China). 5-FU, RAD001, Regorafenib, 17-DMAG and Z-VAD (LC Laboratories, Woburn, MA, USA) were diluted into appropriate concentrations with the cell culture medium.

Cell viability

Cells were plated at 20–30% confluence 24 hours prior to treatment. Unless noted otherwise, cells were treated for 48 hours. Apoptosis was analyzed by nuclear fragmentation assay with 300 or more cells scored for each determination, and by flow cytometry (50,000 events) [3, 4]. Cell proliferation was measured using Cell-Titer 96 Aqueous One Solution Cell Proliferation Assay (MTS assay), according to manufacturers with recommendations. Triplicates were used in each condition, and experiments were repeated for at least 3 times.

Western blotting

Western blotting was performed as previously described (1). The following antibodies were used: Bip (cat#3177, Cell Signaling, Danvers, MA, USA), AKT (S473) (cat#4060, Cell Signaling), AKT (cat#9272, Cell Signaling), eIF4A (cat#ab31217, Abcam), cleaved caspase-3 (cat#9661, Cell Signaling), caspase-8 (cat#9746, Cell Signaling), FoxO1/3 (T24/T32) (cat#9464, Cell Signaling), c-MYC (cat#sc-40, Santa Cruz), $FLIP_{S/L}$ (cat#3210, Cell Signaling), DR5 (cat#ab8416, Abcam), 4E-BP1 (S65) (cat#9451, Cell Signaling), 4E-BP1 (T37/46) (cat#2855, Cell Signaling), 4E-BP1 (cat#9452, Cell Signaling), CHOP (cat#2895, Cell signaling), eIF2a (S51) (cat#3398, Cell signaling), eIF2a (SC-11386), PERK (T980, Cat# 3179), Ki67 (M7240, DAKO), RPS6 (cat#2211, Cell signaling), P70S6K (cat#9234, Cell signaling), Mcl-1 (cat#4579, Cell signaling), Bcl-xL (cat# 20-787-276240, Genway), BID (Cat#2002, Cell signaling), Bax (Cat# SC-903, Santa Cruz), and β-actin (cat#A5441, Sigma).

Transfection

Small-interfering RNA (siRNA) duplexes, and control scrambled siRNA were synthesized from Dharmacon (Lafayette, CO, USA). Cells were transfected with 400 pmols of siRNA duplexes/well in 12-well plates for 4 hours, followed by incubation in medium containing 5% FBS for 20 hours, and then followed by drug treatment in complete media containing 10% FBS. Results are pooled from or average of three-independent wells in a representative experiment, and repeated at least twice with similar results.

Xenograft studies

Nude mice after 1 week of implantation of 4×10^6 HCT116 cells were treated with indicated doses of Episilvestrol, Bortezomib, their combination, or the control buffer (vehicle). Tumor diameters were measured three times a week prior to drug administration, and tumor volumes were calculated ($width^2 \times length \times 0.5$). The averaged tumor volumes of each group were plotted with one standard deviation, and P-value was calculated on the final measurements. Mice were euthanized when tumors reached ~ $800m^3$ in size. Tumors were dissected and fixed in 10% formalin before paraffin embedding. For apoptosis and RT-PCR analysis, three randomly selected

tumor bearing mice were sacrificed to harvest tumors on day 6 for analysis as described [3]. Quantitation was based on sections from two randomly chosen tumors in each group, and scoring of 400 or more cells/tumor in high power fields (400x). Ki-67, cleaved caspase-3, eiF2a (S51) immunostaining were performed on 3 μM paraffin-embedded tumor sections with HRP (Pierce) for signal detection [5].

REFERENCES

1. Zhang L, Yu J, Park BH, Kinzler KW, Vogelstein B. Role of BAX in the apoptotic response to anticancer agents. Science. 2000; 290:989–992.
2. Karbowski M, Norris KL, Cleland MM, Jeong SY, Youle RJ. Role of Bax and Bak in mitochondrial morphogenesis. Nature. 2006; 443:658–662.
3. He K, Zheng X, Li M, Zhang L, Yu J. mTOR inhibitors induce apoptosis in colon cancer cells via CHOP-dependent DR5 induction on 4E-BP1 dephosphorylation. Oncogene. 2016; 35:148–157.
4. He K, Zheng X, Zhang L, Yu J. Hsp90 inhibitors promote p53-dependent apoptosis through PUMA and Bax. Mol Cancer Ther. 2013:2013 Aug 2021. [Epub ahead of print].
5. He K, Chen D, Ruan H, Li X, Tong J, Xu X, Zhang L, Yu J. BRAFV600E-dependent Mcl-1 stabilization leads to everolimus resistance in colon cancer cells. Oncotarget. 2016.
6. Tian X, Ye J, Alonso-Basanta M, Hahn SM, Koumenis C, Dorsey JF. Modulation of CCAAT/enhancer binding protein homologous protein (CHOP)-dependent DR5 expression by nelfinavir sensitizes glioblastoma multiforme cells to tumor necrosis factor-related apoptosis-inducing ligand (TRAIL). J Biol Chem. 2011; 286:29408–29416.
7. Liu X, Yue P, Zhou Z, Khuri FR, Sun SY. Death receptor regulation and celecoxib-induced apoptosis in human lung cancer cells. J Natl Cancer Inst. 2004; 96:1769–1780.
8. Leibowitz B, Qiu W, Buchanan ME, Zou F, Vernon P, Moyer MP, Yin XM, Schoen RE, Yu J, Zhang L. BID mediates selective killing of APC-deficient cells in intestinal tumor suppression by nonsteroidal antiinflammatory drugs. Proc Natl Acad Sci USA. 2014; 111:16520–16525.

Supplementary Table S1: Sequence of Primers and siRNA used in study

Gene	Primer	Sequence
ATF4	Forward	5′-GTTCTCCAGCGACAAGGCTA-3′
	Reverse	5′-GTGTCATCCAACGTGGTCAG-3′
DR5	Forward	5′-AAGACCCTTGTGCTCGTTGT-3′
	Reverse	5′-AGGTGGACACAATCCCTCTG-3′
TRAIL	Forward	5′-GAGCTGAAGCAGATGCAGGAC-3′
	Reverse	5′-TGACGGAGTTGCCACTTGACT-3′
TNFR1	Forward	5′-TGTGCACCTGCCATGCAGG-3′
	Reverse	5′-CAGCACTGTGGTGCCTGAG-3′
CHOP	Forward	5′-TGGAAGCCTGGTATGAGGAC-3′
	Reverse	5′-TGTGACCTCTGCTGGTTCTG-3′
GADD34	Forward	5′-ACTCCCCTAAAGGCCAGAAA-3′
	Reverse	5′-GCTAAAGGTGGGTTCCTGAG-3′
XBP1	Forward	5′-CCTTGTAGTTGAGAACCAGG-3′
	Reverse	5′-GGGTCCAAGTTGTCCAGAATGC-3′
β-actin	Forward	5′-TCACCCACACTGTGCCCATCTACG-3′
	Reverse	5′CAGCGGAACCGCTCATTGCCAATG-3′
PUMA	Forward	5′-CGACCTCAACGCACAGTACGA-3′
	Reverse	5′- AGGCACCTAATTGGGCTCCAT-3′
Noxa	Forward	5′-TTCAGCTCGCGTCCTGCAG-3′
	Reverse	5′-GTTCCTGAGCAGAAGAGTTTGG-3′
Bim	Forward	5′-GGAGACGAGTTTAACGCTTAC-3′
	Reverse	5′-CAAGCAAAATGTCTGCATGG-3′
CALR	Forward	5′-GTTTCGAGCCTTTCAGCAAC-3′
	Reverse	5′-TCTGAGTCTCCGTGCATGTC-3′
CHOP	siRNA	5′-GCACAGCTAGCTGAAGAGA (dTdT)-3′
DR5	siRNA	5′-aagacccttgtgctcgttgtc (dTdT)-3′

Primers were used for RT-PCR analysis. Corresponding sequence (sense) in the cDNA is shown for siRNA. Overhangs are indicated with (). The siRNAs have been extensively used and published by us and others using cancer cell lines from different tissue origins, including CHOP [3, 6] and DR5 [3, 7, 8] .

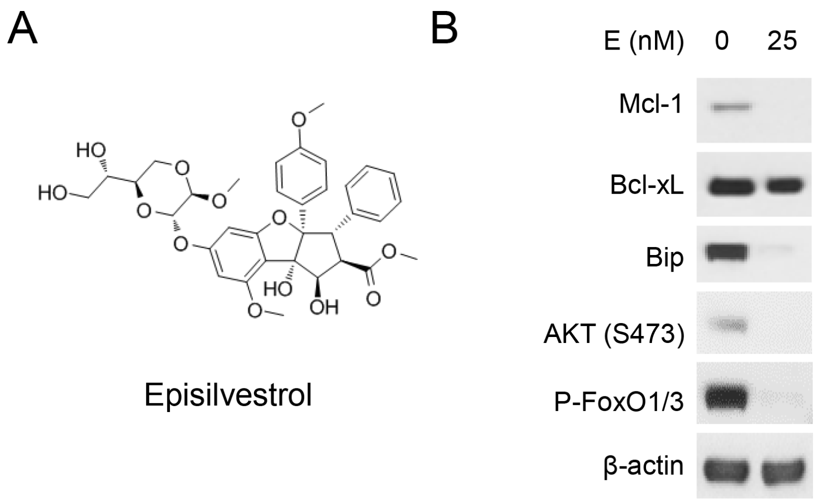

Supplementary Figure S1: The effects of Episilvestrol on HCT 116 cells. (**A**) Chemical structure of Episilvestrol. (**B**) HCT116 cells were treated with 25 nM Episilvestrol for 24 h and analyzed for indicated proteins by western blotting.

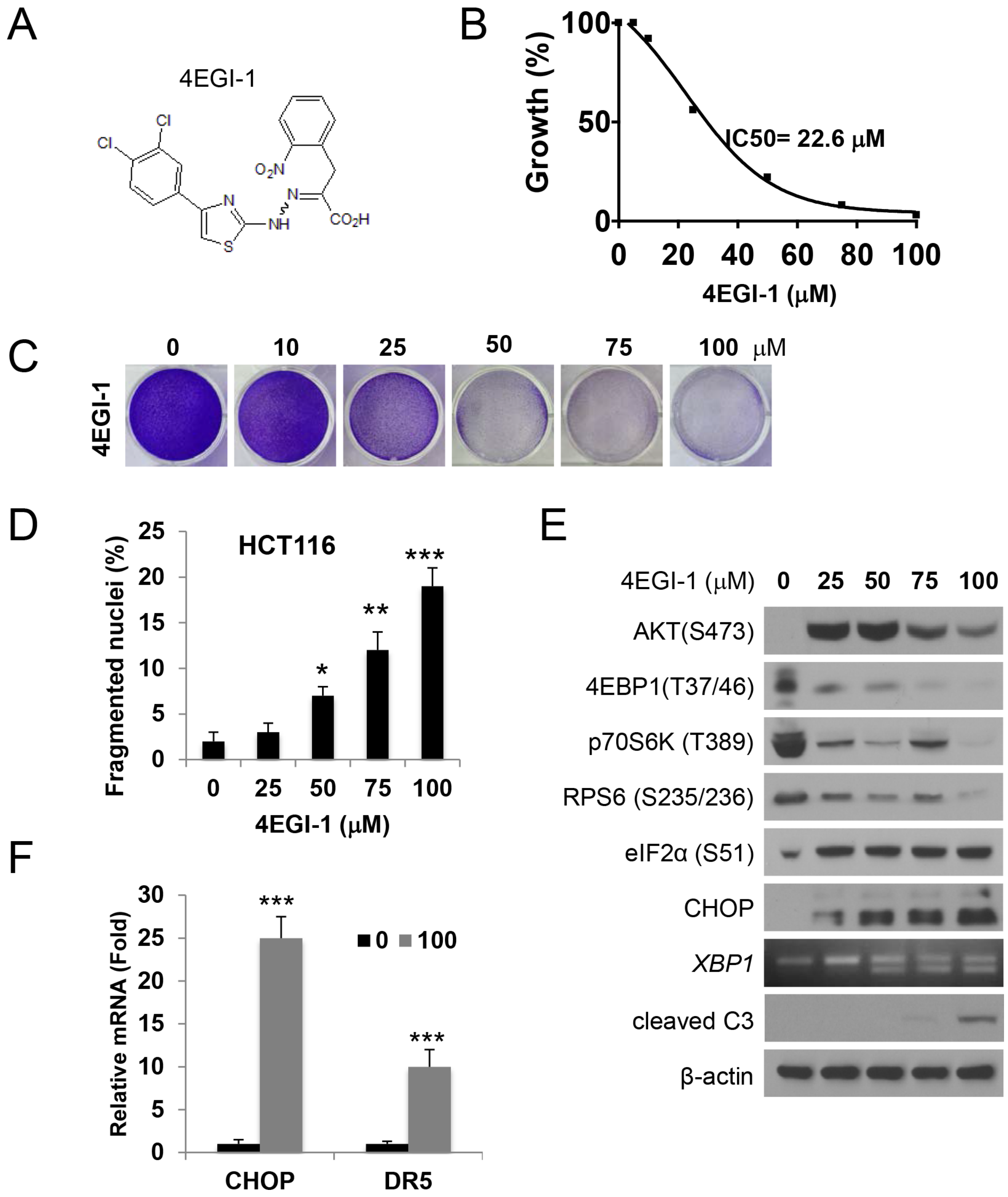

Supplementary Figure S2: ER stress and apoptosis is induced by 4EGI-1 in colon cancer cells. HCT 116 cells were treated with indicated doses of 4EGI-1. (**A**) Chemical structure of 4EGI-1. (**B**) Cell proliferation in 96-wells was measured by MTS assay in triplicates 48 h after treatment. IC50 value was calculated using Prism IV. (**C**) Adherent cells were stained by crystal violet at 48 h. (**D**) Quantification of condensed and fragmented nuclei at 48 h. (**E**) Indicated proteins in cells 24 h after treatment were analyzed by western blotting. (**F**) *CHOP* and *DR5* in cells 24 h after 0 or 100 uM treatment was analyzed by real-time PCR. The levels in vehicle (Un) treated cells were set at 1. D and F, values represent mean+s.d. ($n = 3$). $^*P < 0.05$, $^{**}P < 0.01$, $^{***}P < 0.001$ (Student's *t*-test, two tailed).

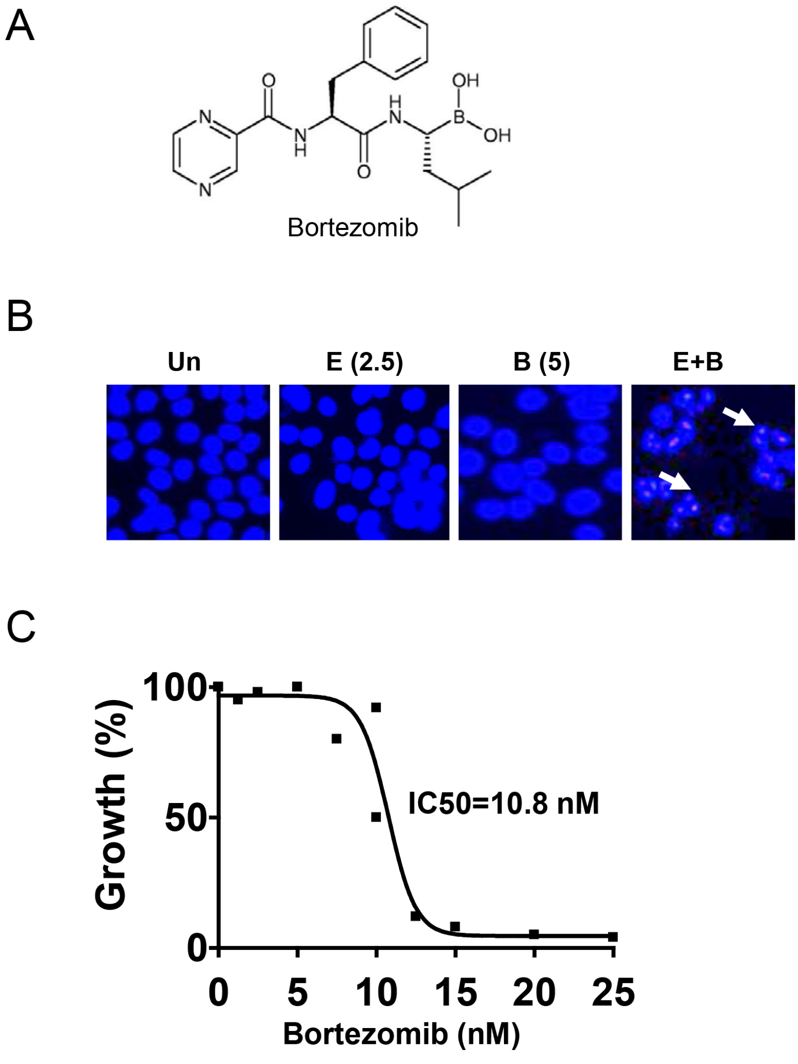

Supplementary Figure S3: The effects of Bortezomib on HCT 116 cells. (**A**) Chemical structure of Bortezomib. (**B**) Representative of nuclei stained with DNA dye Hoechst 48 h after treatment. Arrows indicate fragmented nuclei. (**C**) Cell proliferation was measured by MTS assay in 96-wells 48 h after Bortezomib treatment. IC50 value was calculated using Prism IV.

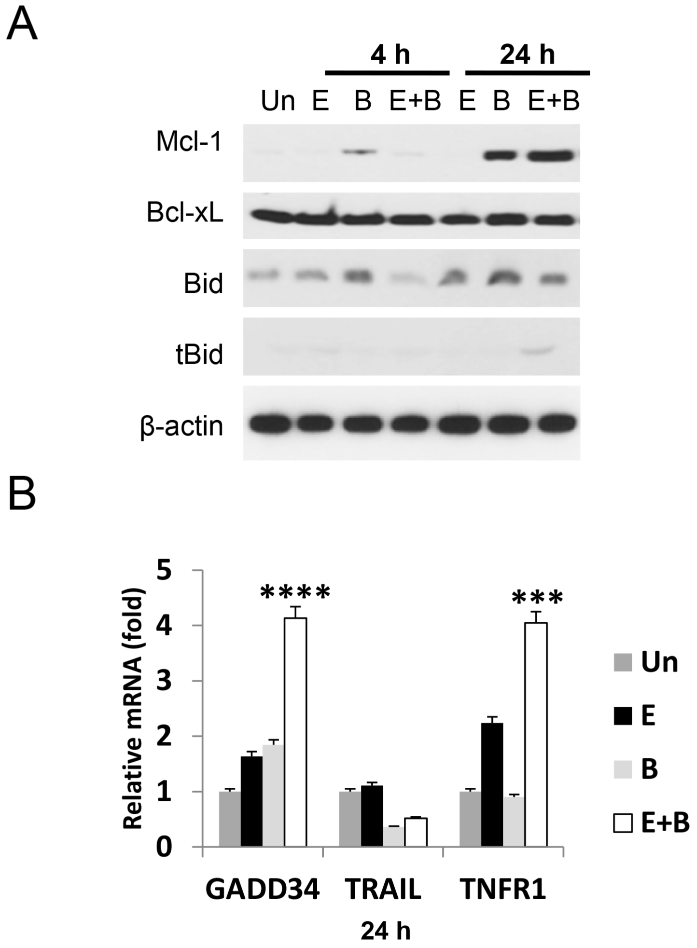

Supplementary Figure S4: Signaling changes induced by Episilvestrol and Bortezomib in colon cancer cells. HCT 116 cells were treated with vehicle (Un), Episilvestrol (E, 2.5 nM), Bortezomib (B, 5 nM), or their combination (E+B). (**A**) The indicated proteins in cells after 4 h and 24 h treatment were analyzed by western blotting. (**B**) Indicated mRNAs were analyzed by RT-PCR. The levels in vehicle (Un) treated cells were set at 1. Values represent mean+s.d. (n = 3). ***P < 0.001, ****P < 0.0001, E+B *vs.* E or B (Multiple comparisons by one way ANOVA followed by Turkey Test).

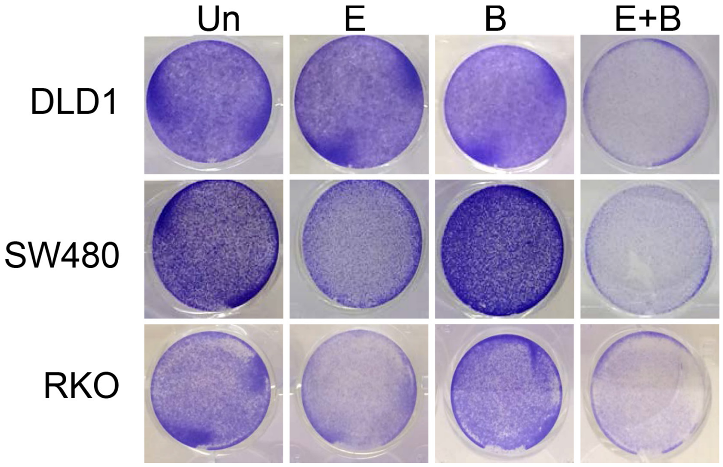

Supplementary Figure S5: Episilvestrol synergizes with Bortezomib in growth suppression of colon cancer cells. Indicated CRC cell lines were treated with vehicle (Un), Episilvestrol (E, 2.5 nM for RKO, and 5 nM for DLD1 and SW480), Bortezomib (B, 5 nM), or their combination (E+B). Adherent cells were stained by crystal violet 48 h after treatment.

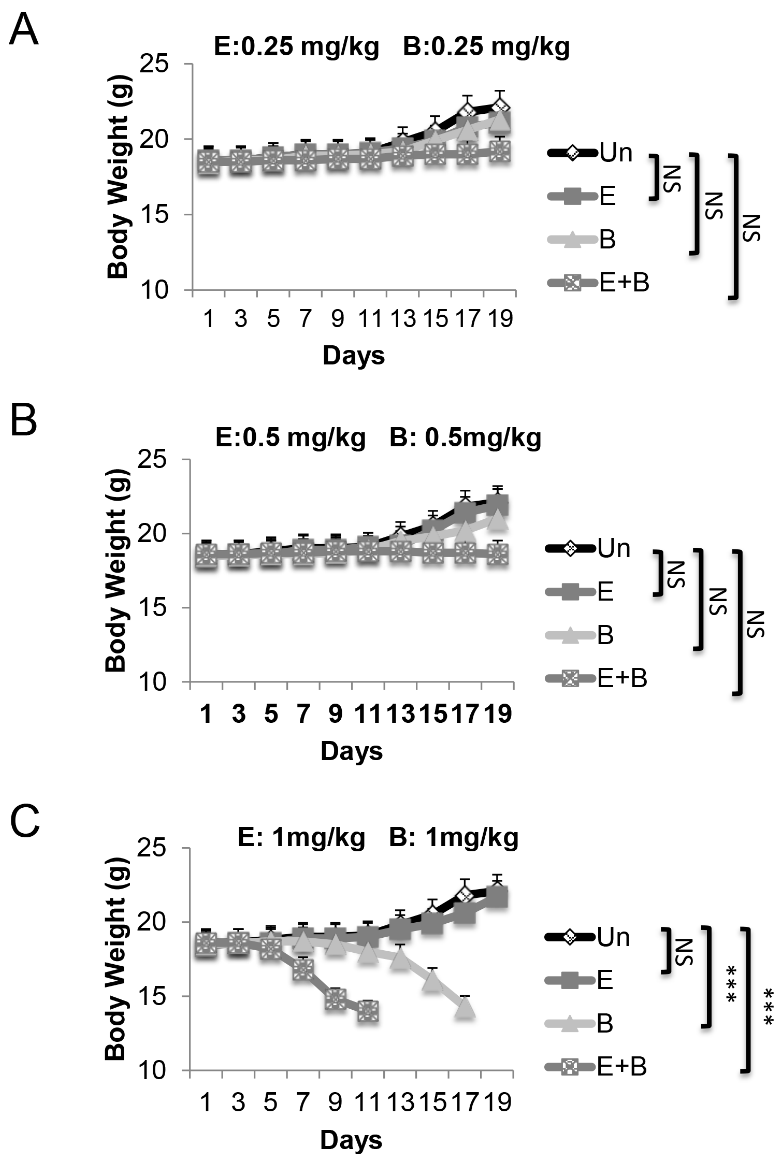

Supplementary Figure S6: Episilvestrol and Bortezomib on body weight. Nude mice bearing HCT116 xenografts were treated with vehicle (Un), or indicated doses of Episilvestrol, Bortezomib, or their combination 3 times a week for a total of 10 times. Body weight was plotted. (**A**) 0.25 mg/kg/D, (**B**) 0.5 mg/kg/D, and (**C**) 1 mg/kg/D. $N = 5$ mice/group. Treatments *vs.* Un. ***$P < 0.001$, (Multiple comparisons by one way ANOVA followed by Turkey Test).
